# An ecophysiologically informed model of seed dispersal by orangutans: linking animal movement with gut passage across time and space

**DOI:** 10.1093/conphys/coy013

**Published:** 2018-03-28

**Authors:** Esther Tarszisz, Sean Tomlinson, Mark E Harrison, Helen C Morrogh-Bernard, Adam J Munn

**Affiliations:** 1 School of Biological Sciences, University of Wollongong, Wollongong, NSW 2522, Australia; 2 Borneo Nature Foundation, Jl. Bukit Raya 82, Palangka Raya 73112, Central Kalimantan, Indonesia; 3 School of Molecular & Life Sciences, Curtin University of Technology, Kent Street Bentley, WA 6102, Australia; 4 Kings Park Science, Department of Biodiversity Conservation and Attractions, Kattidj Close, Kings Park, WA 6005, Australia; 5 School of Geography, Geology and the Environment, University of Leicester, University Road, Leicester LE1 7RH, UK; 6 Centre for Ecology & Conservation, College of Life and Environmental Sciences, University of Exeter, Cornwall Campus, Penryn, Cornwall TR10 9EZ, UK; 7 School of Biological, Earth and Environmental Sciences, The University of New South Wales, NSW 2052, Australia

**Keywords:** ecological service provision, endozoochory, home range estimates, kernel modelling, orangutan, plant–animal interactions, T-LoCoH

## Abstract

Fauna-mediated ecosystem service provision (e.g. seed dispersal) can be difficult to quantify and predict because it is underpinned by the shifting niches of multiple interacting organisms. Such interactions are especially complex in tropical ecosystems, including endangered peat forests of Central Borneo, a biodiversity hot spot and home to the critically endangered orangutan (*Pongo pygmaeus wurmbii*). We combined studies of the digestive physiology of captive orangutans in Australia with detailed field studies of wild orangutans in the Natural Laboratory of Peat-Swamp Forest of Sabangau, Central Kalimantan, Indonesia. By measuring the gut transit time (TT) of indigestible seed mimics (beads) in captivity and applying this as a temporal constraint to movement data of wild orangutans, we developed a mechanistic, time-explicit spatial model to project the seed dispersal patterns by these large-bodied, arboreal frugivores. We followed seven orangutans and established home range kernels using Time Local Convex Hull (T-LoCoH) modelling. This allowed us to model individual orangutan movements and to adjust these models according to gut transit times to estimate seed dispersal kernels. Female movements were conservative (core ranges of 55 and 52 ha in the wet and dry seasons, respectively) and revisitation rates to the same location of *n* = 4 in each 24-h block. Male movements were more unpredictable, yielding fragmented core ranges and revisitation rates to the same location of only 1.2 times each 24 h; males also demonstrated large disjunctions where they moved rapidly over long distances and were frequently lost from view. Seed dispersal kernels were nested predictably within the core ranges of females, but not males. We used the T-LoCoH approach to analyse movement ecology, which offered a powerful tool to predict the primary deposition of seeds by orangutans, thereby providing a reliable method for making *a priori* predictions of seed dispersal dynamics by other frugivores in novel ecosystems.

## Introduction

The ecosystem services provided by animal–plant interactions are complicated and are governed by numerous co-evolved ecological processes (Tylianakis *et al.*, 2010). Consequently, many of these services and their persistence may be sensitive to disruption (McCauley *et al.*, 2012). In many ways, these associations are contingent upon overlaps in the biotic elements of each species’ realized niche (Soberón and Nakamura, 2009), and small changes in the niche and/or behaviour of either the plant or the animal participant can have substantial influences on the other. Consequently, fauna-mediated ecosystem service provision can be highly context-specific, particularly in especially biodiverse systems where there are numerous biotic and abiotic interactions. One such region includes the tropical peat forests of south eastern Asia, which are notably biodiverse and also represent major carbon sinks (Posa *et al.*, 2011). These peat swamp forests support some of the last remaining populations of one of the world’s largest aboreal frugivores, the orangutan. Recent research has strongly supported the role of orangutans as seed-dispersing agents in these peat swamps, and they may be especially important for the dispersal of large-seeded tree species, typical of other tropical forests (Peres and van Roosmalen, 2002; Wotton and Kelly, 2011; Vidal *et al.*, 2013; Donoso *et al.*, 2017; Tarszisz *et al.*, 2017). Consequently, with ongoing pressures of logging and fragmentation, details concerning orangutans and their seed dispersal capacity are much needed.

Animal-mediated seed dispersal, or zoochory, is a crucial component of plant population dynamics, influencing plants and their communities through both short- and long-distance dispersal (Howe and Miriti, 2000; Nathan and Muller-Landau, 2000; Wang and Smith, 2002; Russo *et al.*, 2006; Cousens *et al.*, 2010; McConkey *et al.*, 2012). Zoochory is an important limiting factor for animal-dispersed seeds in several respects. Zoochory can determine the seed deposition location where plants have a potential to establish (Schupp *et al.*, 2010) and may remove the seeds from competition with the parent plant (Howe and Miriti, 2000; Levin *et al.*, 2003; Muller-Landau, 2007; Nathan *et al.*, 2008b; Ruxton and Schaefer, 2012; Schupp *et al.*, 2010), protect seeds from pathogens and predators (Levin *et al.*, 2003; Nathan *et al.*, 2008b; Schupp *et al.*, 2010; Ruxton and Schaefer, 2012) and has the potential to deposit the seeds in beneficial microsites (Nathan and Muller-Landau, 2000; Schupp *et al.*, 2010; Ruxton and Schaefer, 2012). Additionally, in the case of endozoochory, faecal deposition potentially provides a fertilizer (Traveset and Verdú, 2002; Robertson *et al.*, 2006; Traveset *et al.*, 2007; Fuzessy *et al.*, 2016).

The influence of zoochory, and disruptions to this, has recently been powerfully inferred on the basis of population genetic structures of plants, even though their dependence upon zoochory is, in some cases, poorly substantiated (Nathan and Muller-Landau, 2000; Wang and Smith, 2002; He *et al.*, 2009; Krauss *et al.*, 2009; Hamrick and Trapnell, 2011; Pascov *et al.*, 2015), but see Fuzessy *et al.* (2017). There have been advances with respect to connecting seed dispersal studies to movement in recent years, for example with the use of GPS trackers on the animal vectors (Kays *et al.*, 2011; Lenz *et al.*, 2011; Abedi-Lartey *et al.*, 2016; Stevenson *et al.*, 2014), or detailed on-the-ground study of animal movements (Culot *et al.*, 2010; Albert *et al.*, 2013), which this study aims to build on, especially with regard to possible discrepancies between the animal movement and their defecation patterns.

Broadly, movement ecology aims to understand the underlying processes and systems that govern the animal movements in their natural habitats, along with predicting the ecological consequences of those movements (Nathan *et al.*, 2008a; Cagnacci *et al.*, 2010; Hebblewhite and Haydon, 2010; Kie *et al.*, 2010; Morales *et al.*, 2010). A key part of such predictions concerns the spatio–temporal pattern over which animals move, broadly considered as an animal’s home range (HR): the area traversed by an animal in its normal activities of feeding, mating and caring for young, i.e. the entirety of its lifetime (Burt, 1943; Anderson, 1982; Quin *et al.*, 1992). Where we could not follow orangutans for their entire lives, we revert to the concept of a utilization area (Bradshaw and Bradshaw, 2002; Bradshaw *et al.*, 2007) or an occurrence distribution (Fleming *et al.*, 2014), which represents the projection of movement patterns of an animal from observations made for less than an individual’s complete adult life, but which still encompasses enough time to be ecologically relevant. In our interpretation, the seasonal comparisons of male and female orangutans meet this definition best, but for reasons of generality, we still refer to this as HR.

Traditionally, animal HRs have been estimated using location point data to construct minimum convex polygons (MCP) or kernel density estimates (KDE) to define core or larger space-use areas (Laver and Kelly, 2008). However, recent work indicates that the typical MCP methods may overestimate animal space-use and may not adequately reflect patterns of space-use, particularly in complex environments (Getz and Saltz, 2008; Munn *et al.*, 2013). Consequently, predictions of seed dispersal based on traditional metrics of HR may be inaccurate and may not adequately reflect the action of seed-dispersing agents within their habitat.

More recently, two methods have been proposed that more realistically model animal space-use using point data, particularly for complex environments where movements may be governed by physical features, such as that of the largely arboreal orangutan in peat swamps, that of local convex hulls [LoCoH (Getz and Wilmers, 2004; Getz *et al.*, 2007; Lyons *et al.*, 2013)] and the outlier-restricted edge polygons [OREP (Kenward *et al.*, 2008; Munn *et al.*, 2013)] methods. As these approaches are essentially the same, we shall use the term LoCoH hereafter. The sophistication of the LoCoH spatial statistics provide insight not only for where animals go but also how they use space (resources) within their range (Börger *et al.*, 2008; Morales *et al.*, 2010; Jachowski and Singh, 2015), as metrics of time-use such as revisitation and duration of stay are able to be established (Lyons *et al.*, 2013). Consequently, the LoCoH method offers notable advantages for describing the seed dispersal potential of zoochorus agents, which is intricately bound with their movement ecology (Nathan and Muller-Landau, 2000).

In addition to more accurately describing an animal’s movement ecology, the LoCoH methods used herein offer advantages for exploring aspects of animal-mediated seed dispersal, specifically concerning the timing of seed ingestion and elimination in faeces by frugivores, which is an inherently time-based metric. In this regard, the physiology of the dispersal agent can have important consequences for the dependent animal–plant interactions, such as seed dispersal or pollination (Abrol, 2005; McCallum *et al.*, 2013; Seltzer *et al.*, 2013; Tomlinson *et al.*, 2014). The capacity to disperse seeds by endozoochory represents an interaction between the animal, its movement patterns and seed movement from ingestion to elimination, i.e. defecation (Cousens *et al.*, 2010; Fuzessy *et al.*, 2017). Despite a reasonable body of research in other locales, such as the Neotropics (Fuzessy *et al.*, 2017), and past research of orangutans in dipterocarp forests (Galdikas, 1982), there is a paucity of information about present-day orangutan populations (Corlett, 2017) and orangutans in peat swamp forest in general [with the exception of a pilot germination study at the site (Nielsen *et al.*, 2011)]. Our aim is to develop a technique to model home ranges of orangutans that was flexible enough to also estimate their provision of seed dispersal. Understanding and prediction of seed dispersal patterns necessitate an ecophysiologically informed spatial model and our hypothesis was that the increased timescale over which movements were modelled would result in larger seed shadows than predicted by home ranges, but where these shadows would specifically fall was unpredictable. Our ultimate aim in presenting this model is to be able to use it to predict some of the potential alteration of floral diversity in tropical peat swamp, with loss/change of its largest seed dispersal vector, the orangutan. Furthermore, this model could be used to make predictions about the potential impact of orangutans in logged/degraded areas (Morrogh-Bernard *et al.*, 2014; Corlett, 2017).

## Materials and methods

### Captive gut-retention studies

#### Study animals

In total, six orangutans informed these studies: two adult hybrid Sumatran–Bornean orangutans at Taronga Zoo (AEC #4a/11/11), one male (27 years old, 115.5 kg) and one female (29 years old, 66 kg), along with three adult females with infants of varying ages (Female 1: 22 years old, 50.4 kg; Female 2: 24 years old, 40.95 kg; Female 3; 44 years old 42.5 kg) and one adult flanged male (27 years old, 119.6 kg) at Perth Zoo (PZ; AR&E ZA/4991-4 #59404). All animals were fed their regular diet and maintained in their regular enclosures, which consisted of three concrete pens and two separate outdoor areas. Additional banana was added to the regular diet of all the orangutans to hide the seed mimics used (below); additionally, Perth Zoo animals were provided diet cordial (an intermittent dietary ‘treat’). See Supplementary Material for further husbandry details of captive orangutans.

#### Passage times of seed mimics

On Day 1 of each feed trial, the orangutans were each fed different-coloured spheroid, non-toxic polyethylene seed mimics of 2, 4 and 6 mm diameter, with average ± SD masses (*n* = 15 beads per size range) of 22.5 ± 4.6, 28.5 ± 1.5 and 103 ± 2.4 mg (OHAUS Adventurer Analytical, AX423). These seed sizes were chosen as they represent the size range of seeds found intact in faeces from 13 of the wild orangutans followed at the field site during comprehensive studies of the fruits eaten, gut passage of seeds and germination success of gut-passed seeds from orangutans at the field site (Nielsen *et al.*, 2011; Tarszisz *et al.*, 2017). Of note, attempts to disguise larger seed mimics of 8 mm and 10 mm in soft food were unsuccessful. The number of seed mimics ingested by the captive orangutans was comparable with that number of similarly sized seeds found eliminated in the faeces of wild orangutan at the study site (Tarszisz *et al.*, 2017 and see also Nielsen *et al.*, 2011).

Throughout the entire experiment, the orangutans were observed during daylight hours between 0530 and 1730 h. Following ingestion of seed mimics, faeces were collected regularly over 10 days. The enclosure design did not allow for camera placement to observe animals overnight; however, faeces could be distinguished by the presence of different-coloured seed mimics during unobserved times. Faecal elimination in the orangutan is noted as occurring mostly in the morning, with reduced production by afternoon and none overnight (Caton *et al.*, 1999). Preliminary observation of faecal production in wild orangutans agrees with this (Tarszisz unpublished data), and the defecations were observed by the primary investigator (E Tarszisz). When they occurred, however, night samples were considered to have occurred at the midpoint of the sampling interval. Coprophagy was not observed. Faeces were frozen immediately after collection before later thawing to extract eliminated beads.

Faeces were washed through mesh sieves of decreasing diameter (down to 1mm) until all faeces had been examined and all seed mimics collected. As an indicator of the potential seed passage time applied to our mechanistic seed dispersal model, we used the transit time of seed mimics as they first appeared in faeces (i.e. time in hours from ingestion to first appearance in faeces).

### Movements of free-ranging orangutans

The field program was conducted within the Natural Laboratory of Peat-Swamp Forest (NLPSF), a 500 km^2^ area contiguous within the wider 9 200 km^2^ of peat swamp forest in the Sabangau ecosystem, Central Kalimantan, Indonesia (Page *et al.*, 1999; Morrogh-Bernard *et al.*, 2003). This area is managed as part of the multidisciplinary research partnership of the Borneo Nature Foundation (BNF) and their Indonesian counterparts, the Centre for the International Cooperation in Sustainable Management of Tropical Peatlands (UPT LLG CIMTROP) at the University of Palangka Raya. The climate is tropical, with high annual rainfall, separated into distinct wet and dry seasons that last from October to May and June to September, respectively. Daily weather observations at our study site recorded precipitation of 67.31 mm per month on average between October 2012 and May 2013, and 53.13 mm per month on average in June 2013 to September 2013.

Unlike the region’s lowland dipterocarp forests, peat swamp forests such as Sabangau are non-masting and thus produce fruit relatively consistently throughout the year (Cannon *et al.*, 2007a, 2007b). The relative homogeny of the TPSF environment (Singleton and van Schaik, 2001; Singleton *et al.*, 2009), as well as limited secondary seed dispersers (such as rodents) and seed predators (rodents and invertebrates) (D’Arcy and Graham, 2008), makes this an ideal location for modelling overall seed dispersal in TPSF by orangutans because there are likely to be very few effects on dispersal of large seeds other than those related to orangutan movement ecology. Previously home range estimates for orangutans at this site were >560 ha for adult (flanged) male and 250–300 ha for adult females using minimum convex polygons (Morrogh-Bernard *et al.*, 2003, Utami Atmoko *et al.*, 2009) and 1900 ha for males using KDE with the least square cross-validation method (Buckley, 2014). However, these modelling approaches do not allow for deeper interrogation of time–space usage or seed dispersal capacity, and so provide limited capacity for in-depth interpretation of orangutan movements and spatial ecology, let alone of seed dispersal.

### LoCoH methodology

LoCoH uses a non-parametric approach to HR estimation and thereby it circumvents assumptions about the distribution form of the point data that is inherent to parametric kernel methods, thereby reducing HR overestimates (Getz and Wilmers, 2004; Getz *et al.*, 2007; Getz and Saltz, 2008; Munn *et al.*, 2013). This enables that LoCoHs produce a set of non-parametric kernels constructed by aggregating local minimum convex polygons and computing a density estimate distribution for all locations based on nearest neighbour linkages (Getz and Wilmers, 2004; Getz *et al.*, 2007; Getz and Saltz, 2008; Lyons *et al.*, 2013, 2015), the union of which estimates HR (Getz and Wilmers, 2004; Getz *et al.*, 2007).

### Data handling

Focal orangutan follows were conducted following standardized data collection protocols (Martin and Bateson, 1986; Morrogh-Bernard *et al.*, 2002). Activity data (including that of feeding duration and food species) and location data were recorded at 5-min intervals, whereas diet data were collected continuously (Morrogh-Bernard, 2009; Harrison *et al.*, 2010). To ensure that full daily travel patterns were accurately represented, only full-day (nest-to-nest) data were used in this study. Seven individuals, three adult flanged males and four adult females (with juveniles) were followed throughout the study period from October 2012 to December 2013. All point locations were standardized from longitude and latitude into UTM zone 49M coordinates using Earth Point (Clark, 2016) and were transformed into Coordinated Universal Time (UTC) prior to analysis.

Importantly, we have incorporated time into the LoCoH model, described as the T-LoCoH method and incorporates timestamps of each point in both nearest neighbour selection and in the sorting of hulls (Lyons *et al.*, 2013). Of three possible methods, we pursued the *a*-LoCoH method, which reduces the number of nearest neighbours in areas with thin, scattered points, to better homogenize potential sampling bias. The ‘*a’* method adds cumulative distance from the parent point up to an ‘*a*’ value and determines nearest neighbours whose aggregate distance is ≤ *a* (Lyons *et al.*, 2013, 2015) and can be superior to other T-LoCoH methods for reducing the minimum spurious hole covering (Getz *et al.*, 2007; Lyons *et al.*, 2013).

Since time is a critical factor contributing to space usage in T-LoCoH, the first step is to determine an appropriate value by which to scale the maximum theoretical velocity, denoted by Lyons *et al.* (2013) as the dimensionless factor *s*. To construct the home range kernels of the orangutans, we selected 24-h intervals because orangutans are largely diurnally active, sleeping from dusk to dawn (Mitra Setia *et al.*, 2009). When modelling seed dispersal hullsets, *s* was chosen based on the transit time for seed mimics determined in captive orangutans. As there was no significant difference in gut passage times between seed mimic sizes of 2, 4 and 6 mm (see below), an average passage time of 76 h was applied as the intervisit gap (IVG). To project the largest possible seed dispersal kernels, we also used a maximum passage time of 133h as the IVG.

#### Kernel model refinement

In applying the *a-*LoCoH approach, the most appropriate value of *a* was established by examining the differing density of isopleths, overlaid on GIS data to reduce both type I (including areas that are not part of the home range) and type II (overlooking areas that are part of the home range) errors. We checked the validity of the initial value of *a* by visually assessing whether the ‘*a*^th^’ isopleth encompassed 95% of the data, which is often used as definition of the home range (Laver and Kelly, 2008; Lyons *et al.*, 2013). We used the relationships between isopleth area and edge:area ratios and *a* to determine the least erroneous values for each individual’s movement patterns following the guidelines suggested by Lyons *et al.* (2013). As each animal had a different *V*_max_, which was the maximum observed velocity between any two consecutive points, and different movement patterns, the *a* value differed between each animal.

### Temporal effects

We computed revisitation rate (the number of visits to the same GPS location) and duration of use by first specifying an IVG of 24 h. This means that observations were only recognized by the T-LoCoH model as separate visits if at least 24 h had elapsed between them. Secondly, an IVG of 76 h, the average time for a transit of an undigested seed and an IVG of 133 h, the longest transit time for a seed, were specified, creating metrics for revisitation and duration of use over these larger time scales. In effect, for each individual, we modelled two ‘animals’ separately: the orangutan that moved in ‘real time’ and the average seed in their gut passage, which was approximately three times ‘slower’ on average, and over five times ‘slower’ at its slowest. Seed dispersal was therefore explored by interrogating the differences in revisitation, duration of stay and space-use between these first and second ‘animals’.

**Figure 1:**
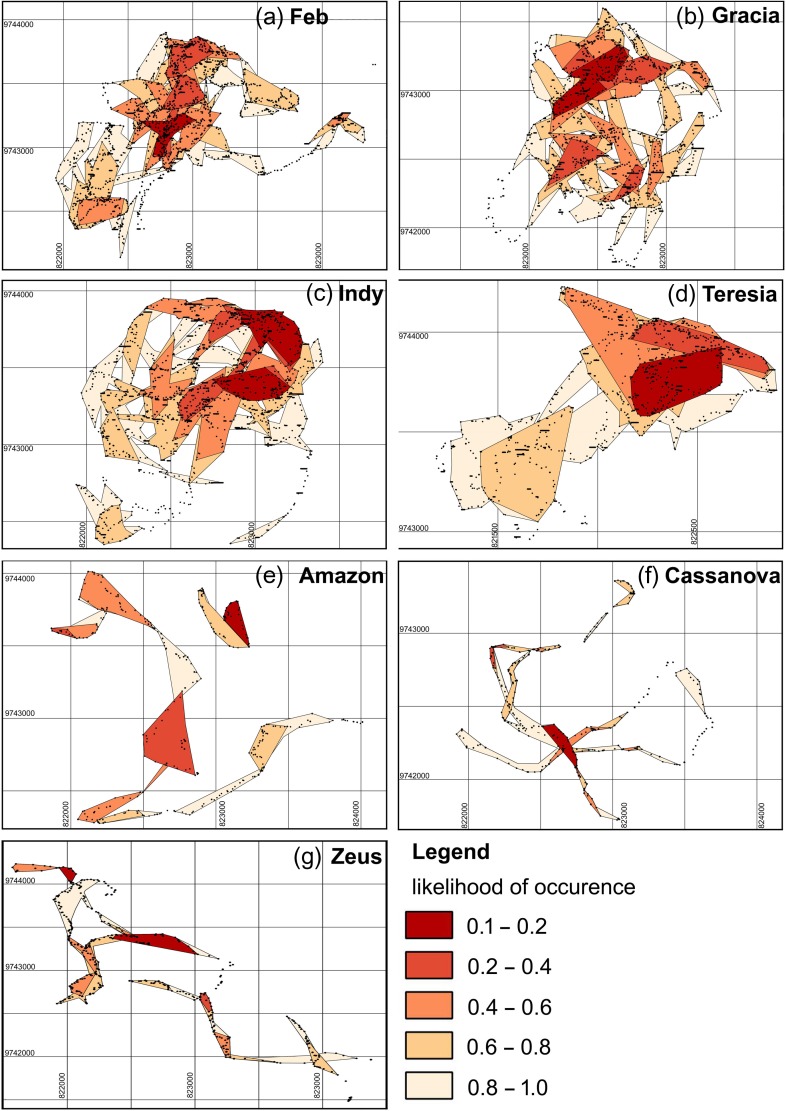
Likelihood distribution kernels and revisitation points (dots), as determined by T-LoCoH analysis period = 24 h for females (**a–d**) and for males (**e–g**)

Spatially explicit projections (Fig. 1) were generated by exporting the probability kernels as shape files and displaying them using the GIS package qGIS v2.4.0-Chugiak. The home range estimates resulting from the T-LoCoH approach were compared against MCP estimates computed using the ‘convex hulls’ command in qGIS that is consistent with previous studies of orangutan home ranges at NLPSF (Morrogh-Bernard *et al.*, 2003; Utami Atmoko *et al.*, 2009; Buckley, 2014).

### Statistical analyses

In order to test the capacity of the kernel models to predict defecation, known locations of defecation were recorded in the field and not used to train the model. These locations were intersected with the kernel models of defecation/seed dispersal in qGIS. The expected proportions of defecation points falling into each kernel were tested against the observed proportion falling into each kernel using Pearson’s chi-squared test for all animals, and also for males and females only.

We explored the effect of sex and season on orangutan movements and seed dispersal capability by constructing generalized linear models (GLMs) of several modelled elements of orangutan movement, including step length, 80% kernel area, residency (revisitation rate) and duration of stay. Step length refers to the use of the Pythagorean Theorem to calculate the Euclidean step length distances between subsequent GPS fixes.

The core range was initially defined by examining the distribution of hulls in time-use space, choosing a value of *a* that filled core areas and minimized spurious crossovers (Lyons *et al.* 2013) and is defined here as the 20% likelihood kernel (i.e. 80% kernel area). This describes locations that are the most heavily used, which encompass a small proportion of known locations.

Tests were constructed using a fully factorial design of sex and season. All analyses were conducted using R v3.2.2 [R Core (R Core Team, 2015)] in the R studio shell v0.99.48 (R Studio Team, 2015), and all data are reported as means ± 1SEM unless stated otherwise.

## Results

### Gut passage times

From our captive feeding trials, the TTs were of 70.6 ± 7.1, 72.5 ± 6.8 and 86.2 ± 16.6 h for the 2, 4, and 6 mm seed mimics, respectively. The maximum TTs were 159.3 ± 14.2, 118 ± 18.4, and 118.0 ± 19.6 h for the the 2, 4, and 6 mm seed mimics, respectively. (see Supplementary Material for further details). There were no significant differences in the TTs between any seed mimic (Supplementary Material, *F* = 0.36, d.f. = 2; *P* = 0.54), and the common TT_max_, averaged 133 h, while the average TT for all seed mimics was 76 h.

### Orangutan movement ecology

Kernel models showed that, with the exception of two related females, the focal orangutans were semi-solitary, with very little overlap between models of the same sex. Males tended to have much more disjunct movement patterns than females and also tended to overlap several females’ home ranges within their own. Total female orangutan home ranges overlapped by 21.9 ± 11.2 ha, heavily influenced by the large degree of overlap between two related females (approximately 65 ha). Total male orangutan home ranges overlapped by 3.4 ± 1.9 ha. The total home ranges of our focal males overlay the home ranges of all our focal females, averaging 216.9 ± 9.2 ha overlap.

Home ranges were characterized by significantly higher revisitation rates for females (4.01 ± 0.02 visits per day for females compared to 1.24 ± 0.01 visits per day for males, see Table 1) in the core range. Females furthermore had long loops of short duration and low revisitation around the edges of their home ranges. This pattern appeared potentially true for the males, but the data were not extensive enough to state this definitively.

**Table 1:** Effects of season and sex on the measures of orangutan movement at NLPSF extracted from T-LoCoH kernel modelling.

		**Step length (m)**			**Revisitation rate**			**Duration of visit**		
		Mean (S.E.)	*F*_1,10_	*P*	Mean (S.E.)	*F*_1,10_	*P*	Mean (S.E.)	*F*_1,10_	*P*
**IVG = 24 h**										
Season	Dry	8.61 (0.15)	4.23	0.0667	3.37 (0.02)	0.001	0.974	43.06 (0.30)	0.344	0.571
	Wet	9.12 (0.16)			3.48 (0.02)			39.29 (0.21)		
Sex	M	7.28 (0.26)	13.1	**0.00474**	1.24 (0.01)	70.9	**7.51 × 10**^**−6**^	61.69 (0.40)	22.0	**8.51 × 10**^**−4**^
	F	9.32 (0.12)			4.01 (0.02)			35.50 (0.18)		
Season×sex	DM	6.34 (0.39)	0.001	0.997	1.18 (0.01)	0.0167	0.900	65.57 (0.68)	0.323	0.582
	WM	7.90 (0.35)			1.27 (0.01)			59.16 (0.47)		
	DF	8.57 (0.17)			3.85 (0.02)			38.03 (0.30)		
	WF	9.98 (0.18)			2.05 (0.02)			33.26 (0.19)		
**IVG = 76 h**										
Season	Dry	–			2.07 (0.40)	2.96	0.123	54.9 (7.17)	4.56	0.0653
	Wet	–			1.53 (0.18)			69.9 (6.67)		
Sex	M	–			1.00 (0.00)	11.2	**0.0102**	79.1 (0.73)	11.2	**0.0100**
	F	–			2.17 (0.24)			54.1 (4.71)		
Season×sex	DM	–			1.14 (0.14)	0.809	0.395	72.7 (15.1)	0.052	0.825
	WM	–			1.23 (0.01)			85.5 (2.67)		
	DF	–			2.53 (0.42)			46.0 (3.27)		
	WF	–			1.80 (0.10)			62.1 (7.02)		

Revisitation rate here is the number of visits to the same location per 24 h and the duration of visit gives the average number of minutes spent at each location. Note: Step lengths were not directly calculable for seed dispersal estimates at IVG *=* 76h

. Bold *P*-values are statistically significant at *P* < 0.01.

The core range was initially defined by examining the distribution of hulls in time-use space, defined here as the 20% likelihood kernel, describing locations that are the most heavily used. The hullsets that resulted for male orangutans were highly fragmented as a result of their disjointed movements (Fig. 1), yielding small and fragmented 20% likelihood kernels. The average home range size estimated for a female orangutan at NLPSF by T-LoCoH was 55.2 ± 12.00 ha, with an average step length of 8.89 ± 0.11 m, a revisitation rate of 3.43 ± 0.02 visits each day and average visit duration of 41.00 ± 20.18 min. The T-LoCoH home range estimated for females in the dry and wet seasons were 55.31 ± 6.97 ha and 52.38 ± 8.35ha, respectively. The minimum convex polygons for females in the dry and wet seasons were 149.00 ha and 160.84 ha, respectively.

There were differences in all of the movement parameters of the orangutans between the sexes, while season had no influence. Sex had a significant effect on all indices including the step length (*F*_1,10_ = 13.0; *P* = 0.0047), revisitation rate (*F*_1,10_ = 70.9; *P* = 7.51 × 10^−6^) and duration of visit (*F*_1,10_ = 22.0; *P* = 8.50 × 10^−4^). For the 80% kernel area, the influence of sex was significant (*F*_1,5_ = 16.78; *P* = 0.009), although season did not significantly influence the home range area of females (*F*_2,5_ = 0.70; *P* = 0.540). Revisitation rates were higher and intervisit duration shorter for females than for males in both seasons (Table 1).

### Seed dispersal projections

**Figure 2:**
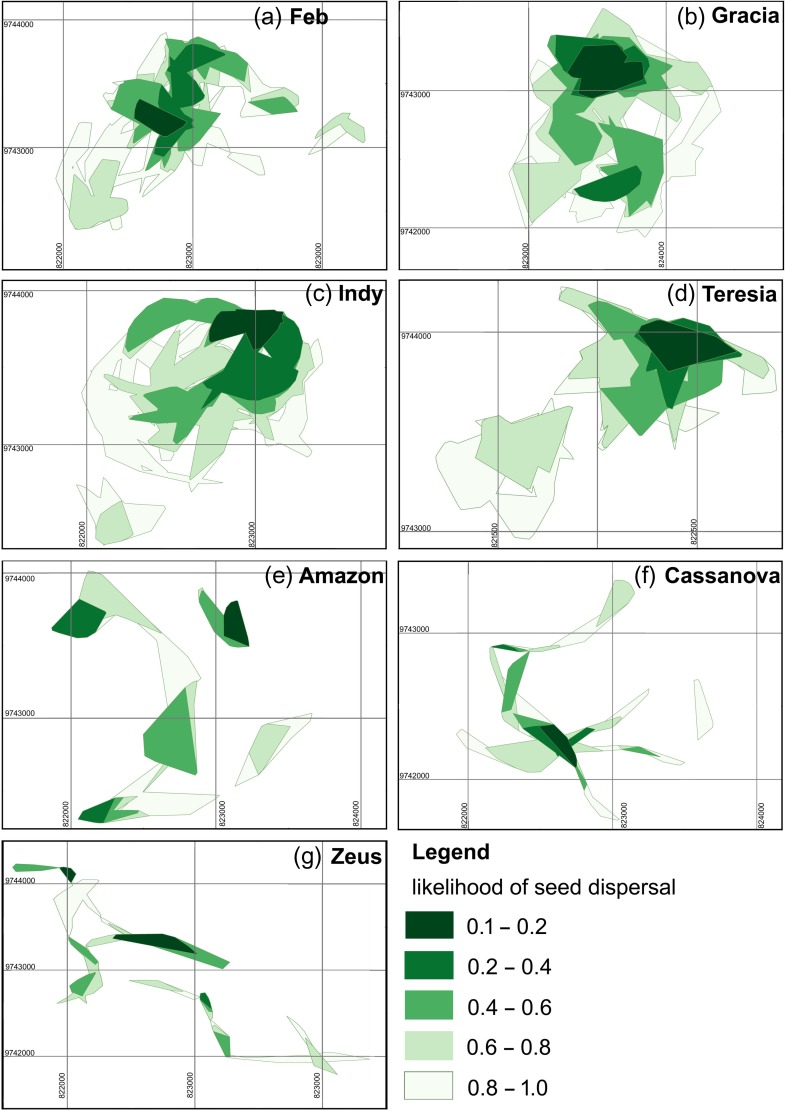
Seed shadow kernels projected by T-LoCoH at analysis period = 76 h estimated as the average gut passage time of seeds by orangutans. Generally, most likely seed shadow is more localized than home range for females (**a–d**), but becomes much less predictable for males (**e–g**).

The average 76-hr 80% seed shadow estimated for a female orangutan at NLPSF by T-LoCoH was 52.4 ± 6.44 ha (Fig. 2), with an average revisitation rate of 2.17 ± 0.244 visits every 76 h. The T-LoCoH seed shadow estimated for females in the dry and wet seasons was 57.3 ± 10.46 ha and 47.5 ± 8.24 ha, respectively. The average 133-hr 80% likelihood seed shadow estimated for a female orangutan at NLPSF by T-LoCoH was 94.2 ± 7.49 ha. Due to their disjunct movement patterns, the seed shadows projected for males were much less certain and could not be projected for all individuals beyond 76 h, nor in all seasons. The average 76-hr 80% seed shadow estimated for a male orangutan at NLPSF by T-LoCoH was 17.3 ± 3.93 ha (Fig. 2)

When the seed dispersal kernels projected at the average 76-hr gut passage time were analysed, sex alone influenced the area of the seed shadows (*F*_1,8_ = 11.4; *P* = 0.0097), revisitation rate (*F*_1,8_ = 11.2; *P* = 0.0102) and duration of stay (*F*_1,8_ = 11.2; *P* = 0.010). Season became marginally significant for duration of stay only (*F*_1,8_ = 4.56; *P* = 0.065). Seeds were likely to be dispersed over a larger area by females, but revisitation was higher, and duration of stay shorter; this was also the case across both seasons (Table 2a,b). Essentially, dispersal by males resulted in smaller, more discrete dispersal ‘islands’ than dispersal by females because their movement patterns were projected over longer, narrower corridors (Fig. 2).

**Table 2:** Effects of sex-season concatenate on revisitation rate for WF = female wet season, DF = female dry season, WM = male wet season, DM = male dry season.

	Group 1	Group 2	Difference	Adjusted *P* value
**A) IVG = 24h**				
WF—DF	2.05 ± 0.02	3.05 ± 0.02	0.293	**<0.01**
DM—DF	1.18 ± 0.01	3.05 ± 0.02	−2.268	**<0.01**
WM—DF	1.27 ± 0.01	3.05 ± 0.02	−2.58	**<0.01**
DM—WF	1.18 ± 0.01	2.05 ± 0.02	−2.97	**<0.01**
WM—WF	1.27 ± 0.01	2.05 ± 0.02	−2.873	**<0.01**
WM—DM	1.27 ± 0.01	1.18 ± 0.01	0.096	0.344
**B) IVG = 76h**				
WF—DF	3.87 ± 0.02	3.42 ± 0.02	0.444	**<0.01**
DM—DF	1.16 ± 0.01	3.42 ± 0.02	−2.261	**<0.01**
WM—DF	1.23 ± 0.01	3.42 ± 0.02	−2.19	**<0.01**
DM—WF	1.16 ± 0.01	3.87 ± 0.02	−2.705	**<0.01**
WM—WF	1.23 ± 0.01	3.87 ± 0.02	−2.633	**<0.01**
WM—DM	1.23 ± 0.01	1.16 ± 0.01	0.071	0.522

Data are presented as mean ± S.E.M. Adjusted *P* values represent the probability of differences offset against the effects of multiple comparisons, representing the smallest ‘family’ error rate at which the null is rejected. Bold *P*-values are statistically significant at *P* < 0.01.

### Model validations

There was no significant difference between the proportion of defecation events observed in each seed dispersal kernel and the likelihood of seed dispersal predicted by the model projections (Pearson’s *χ*^2^_5_ = 8.09; *P* = 0.151). The mean average percent error (MA%E) of model predictions was 3.86 ± 0.97 %, ranging from 1.05% to 7.89%. The model fit was stronger for females only (*χ*^2^_5_ = 0.229; *P* = 0.999), but marginally less so for males only, although they were not statistically significant (*χ*^2^_5_ = 8.28; *P* = 0.141).

## Discussion

To the best of our knowledge, this is the first study to incorporate a time constraint in the construction of kernels, thereby presenting the first ecophysiologically informed kernel models to predict the spatial consequences of animal–plant interactions via seed dispersal. Importantly, the diet of the wild orangutans observed, from this and earlier studies (Morrogh-Bernard *et al.*, 2009), was comparable with that of the captive animals used to measure the seed mimic gut passage times, in that fruits and some vegetation made up the bulk of the orangutan diet. As such, we are confident that the seed mimic TTs that we measured from captive animals presents realistic temporal constraints defining the likely seed dispersal patterns by the wild animals, at least within the peat swamp habitat reported here. Overall, only sex influenced the measures of orangutan movement, and males tended to move further and more erratically than females. The data that we collected for males, however, were less consistent than for females because we were less able to repeatedly find and follow the males, reducing our confidence in the analysis of their data. Females tended to spend all their time foraging, likely moving mainly in search of fluctuating food resources typical of peat swamp forest (Cannon *et al.*, 2007a, 2007b), while male movements may be more motivated by the search for mating opportunities. Kernel models constrained on the basis of gut transit times resulted in a longer time interval, implying a ‘slower’ rate of movement for seeds than for their orangutan dispersal vectors. To the best of our knowledge, the only other study to investigate feed passage rates in orangutans was by Caton *et al.* (1999). However, Caton *et al.*’s (1999) study examined the gut passage of small particle (size) and fluid markers; thus, we felt that our study had merit as we are primarily interested in the passage of larger indigestible seed markers in order to extrapolate reliable information on seed passage relevant for broader scale seed dispersal studies. Of note, the seed mimic elimination patterns were more staggered and less smooth than the typical elimination pattern of finer particles and, combined with the much smaller number of seeds typically ingested, the standard measures of particle MRT may not adequately describe seed passage patterns.

### Orangutan movement, sexes and seasons

The MCP estimates of home range that we generated for females in each season (150 ha in the dry and 160 ha in the wet) are consistent with previous reports at this study site (Morrogh-Bernard, 2009; Singleton *et al.*, 2009). MCP estimates for males were even larger due to their greater and more erratic movement patterns, consistent with reports by Buckley (2014), who followed orangutans in the same location from 2010 to 2012. Although our MCP home range estimates more closely approximated previous findings, our T-LoCoH estimates are approximately 36% of our MCP projections for females across both seasons. Of note, the kernel areas we have described (Fig. 1) gave an integrated time–space view of orangutan home range use for females, as opposed to previous kernel areas based on space alone (Morrogh-Bernard, 2009; Singleton *et al.*, 2009). As a result, these more precise estimates produced home ranges that were, on average, 10% of the previously published estimates at NLPSF (Morrogh-Bernard, 2009; Singleton *et al.*, 2009; Buckley, 2014). Large discrepancies between LoCoH methods and more traditional methods (MCP, KDE and alpha-hull) have been reported in other studies (Getz and Wilmers, 2004; Getz *et al.*, 2007; Munn *et al.*, 2013). LoCoH approaches tend to produce smaller, more refined estimates than MCP or KDE with fewer type I and II errors (Getz and Wilmers, 2004; Getz *et al.*, 2007; Munn *et al.*, 2013), and our data further confirmed that traditional home range methods such as MCP can substantially overestimate home range and space-use. The incorporation of time aims to take the concept of home range from a static spatial construct, such as the MCP where all known locations are considered equally, towards a more realistic evaluation of space-use, weighting areas where greater time is spent with greater importance.

The use of T-LoCoH generated several informative parameters that described the movement ecology of orangutans at NLPSF: kernel area, revisitation rate, step length and duration of stay. Revisitation rates and duration of stay can illustrate the importance of different locations between sexes. Our integration of time has shown significant interactions between both how and where space is used between sexes (Table 1). The movement parameters generated by T-LoCoH (step length, revisitation rate and duration of stay) for orangutans at the NLPSF were all influenced by sex and all suggested that males ranged over greater areas than females, but were resident for less time, and visited each location less often than females, similar to previous studies (Utami Atmoko *et al.*, 2009). Females had more predictable movement patterns within a more structured core area, and from this, we infer that the females were most likely moving order to meet their ecological energetic requirements.

We also did not detect any seasonal patterns in the movements of male orangutans, and their more unstable core ranges suggested that they had more fluid home ranges that did not fluctuate in accordance with patterns of fruiting at NLPSF. Rather than moving principally or only to forage, males were potentially moving in relation to another powerful imperative—that of mating and/or avoiding (or aggressing) other conspecifics, as Utami Atmoko et al. (2009) have suggested previously.

By modelling the movements of orangutans using T-LoCoH, and specifically incorporating different time and space-use metrics to estimate behaviour patterns, we have both refined the projected home ranges and uncovered possible differences in the motivations of habitat use between males and females. These models are replicable for other individuals and can be readily remodelled as additional data are gathered at the study site (of BNF/CIMPTROP) in ongoing orangutan monitoring projects. Furthermore, due to the malleability of this model, we have been able to extend this to the prediction of downstream ecological patterns resulting from orangutan movement in the form of their likely seed dispersal activity.

### Implications for predicting seed dispersal

When temporally constrained on the basis of known gut transit of seeds by orangutans, the dispersal kernels created were similar to the 24-h movement kernels of the orangutans themselves, but the ‘seed kernels’ at 76 h are more likely to ‘travel’ through a circuit of the home range and return (or rather, be deposited) in the core utilization area. Essentially, projected defecation points were more closely distributed in space, clustering more closely within the core home range of the focal orangutan, particularly for females, which had the more predictable movement patterns. Primary endozoochorous seed dispersal can be effectively predicted on the basis of where an animal, in this case an orangutan, will defecate (Wang and Smith, 2002; Cousens *et al.*, 2010). Our model predictions of defecation patterns were well supported by the *χ*^2^-test of actual defecation data, with only a small (<10%) error, suggesting that physiologically informed T-LoCoH models should provide accurate estimates of primary seed dispersal.

The movement of seeds can powerfully contribute to tree species’ colonization, succession and post-disturbance recovery, and consequently therefore ecological restoration and management (Wang and Smith, 2002; Bascompte and Jordano, 2007; Schupp *et al.*, 2010; Ruxton and Schaefer, 2012; Côrtes and Uriarte, 2013). Seed dispersal also represents half of the gene flow pattern of plant populations [the other half being pollination (Abrol, 2005; Krauss *et al.*, 2009; Menz *et al.*, 2011; McCallum *et al.*, 2013)], and so is a powerful contributor to population genetic structure. As a critical element of ecological and evolutionary processes, the mechanistic estimation of passive seed dispersal has made considerable strides (Wright *et al.*, 2008, Nathan *et al.*, 2011, 2002). The modelling of plant–animal interactions in a mechanistic manner has, however, remained somewhat elusive, with most zoochory studies applicable only to the time and place of their model training (Cousens *et al.*, 2010; Schupp *et al.*, 2010; Côrtes and Uriarte, 2013). This is largely due to the plethora of stochastic influences on zoochory, such as sex, season, reproductive patterns and ecological energetics (Nathan *et al.*, 2008a), all of which make prediction of animal movements difficult, even in a hypothetically stable ecological system (Guisan and Zimmermann, 2000; Guisan and Thuiller, 2005). In novel ecological ‘hyperspace’ represented by areas of changing land-use and/or climate, the changing patterns of ecological cascades that influence spatial population structure are rendered unpredictable (Dormann *et al.*, 2012; Mesgaran *et al.*, 2014). Thus, while we have developed a unique set of mechanistically informed models of likely seed dispersal patterns for the NLPSF, extrapolating from these into different tropical peat forests or into other orangutan habitats, such as dipterocarp forest, may require further model training. Nonetheless, this study firmly demonstrates how movement and gut transit times of female orangutans influence seed deposition shadows. Furthermore, it suggests that seed dispersal by female orangutans is linked to their foraging activity and that their movement and seed dispersal patterns will change in relation to food availability. This has potentially serious implications for forest structure and genetic isolation if the habitat is disturbed or population levels decrease, particularly for the large-seeded tree species they were found to have endozoochorously transported (Tarszisz *et al.*, 2017).

### Limitations of this study

Time and logistical constraints made continuous monitoring of the same animals difficult, perturbing the internal consistency of our data. In particular, there is a paucity of data on adult males, compared with adult females, due to their increased space-use requirements (Utami Atmoko *et al.*, 2009, Buckley, 2014), their fast movement on the ground, causing increased ‘loss’ of males during follows compared to females, and their more labile home ranges, based on competition with both flanged and unflanged males. While these home range models are partially indicative of male orangutan movements, they do not give as complete or refined a picture as emerges for the females. It is entirely possible that with more data for males, we may have found some stability and connectivity of male home ranges. Although Buckley (2014) addressed some of these issues, the consistency of our results with previous research suggests that this problem is a general constraint on the orangutan movement ecology literature (Utami Atmoko *et al.*, 2009). Affixing remote sensing (GPS tags) could have facilitated data collection without any risk of the presence of human observers disturbing the orangutans and altering their movements. Remote sensing would also guarantee consistent survey effort, regardless of the constraints of manpower and inclement conditions (Kie *et al.*, 2010; Tomkiewicz *et al.*, 2010; Lyons *et al.*, 2013; Munn *et al.*, 2013). However, the application of different technology must be considered in the light of other data that would be lost in remote sensing, such as defecation locations and feeding observations, in addition to ethical and logistical issues.

The timescale we followed orangutans was only a relatively short period, when compared with their life history. Longer observations could yield more accurate ranging and space-use data and should produce more accurate models. Furthermore, continual incorporation of faeces location should yield more accurate data for seed dispersal. This would provide the opportunity to build on the data we have collected here.

### Future directions

A T-LoCoH approach appears to provide a method to accurately predict (estimate) orangutan movement within TPSF, and we suggest that it is likely that seed dispersal cascades will be similar in other TPSF landscapes, both within and outside of Sabangau. TPSF is an important orangutan habitat that is considerably less studied than the region’s dipterocarp forests, although this has begun to be redressed in recent years (Rieley *et al.*, 1997; Page *et al.*, 1999, 2011; Jauhiainen *et al.*, 2005; Harrison *et al.*, 2010; Hooijer *et al.*, 2010; Morrogh-Bernard *et al.*, 2011, 2014; Beaudrot *et al.*, 2013). Ecological processes may differ considerably between TPSF and non-peat tropical forests (Cannon *et al.*, 2007a; Harrison, 2013), and the ultimate goal of a modelling approach should be the generation of models that can produce context-specific projections that capture these differences.

Our models of orangutan movement and seed dispersal provide projective capacity for novel locations or ecosystems by being data-referential. While our model was not completely static, allowing for extrapolation to other TPSF areas, the next step towards a fully predictive model would be using such models to identify training areas, overlaying T-LoCoH models with mechanistic niche envelope estimates (Austin, 2007; Kearney and Porter, 2009; Kearney *et al.*, 2010, 2012; Mesgaran *et al.*, 2014), making it possible to project orangutan movements and seed dispersal without *a priori* expectations in novel habitats.

A major criticism of modelling focused research programs is that the model represents a set of evidence-based hypotheses that are rarely tested (Tomlinson *et al.* 2014). Our internal statistical tests notwithstanding, it should be noted that we have not provided any empirical tests of our model hypotheses here. The modelling of seed dispersal, whilst being a process that contributes to the population structures of the plants dispersed (McConkey, 2000; Wang and Smith, 2002; Jordano *et al.*, 2007; Cousens *et al.*, 2010; Côrtes and Uriarte, 2013), and the community that results (Howe and Miriti, 2000; Wang and Smith, 2002; Bascompte and Jordano, 2007; McConkey *et al.*, 2012), is also a model prediction of plant maternal gene flow (Wang and Smith, 2002; Jordano *et al.*, 2007; Hamrick and Trapnell, 2011). This implies that measurements of plant maternal gene flow could be used to test these models. These could be carried out using parentage assignment of seeds collected from orangutan defecation within the bounds of the models constructed herein, using an array of emerging next-generation sequencing technologies (Pritchard *et al.*, 2000; Chen *et al.*, 2007; Poland *et al.*, 2012; Grabowski *et al.*, 2014).

## Conclusions

- Our data provide a mechanistic link between animal movements and the provision of endozoochory. The approach offers a powerful tool to reliably begin predicting the primary deposition of seeds by a large-bodied species such as the orangutan in contiguous TPSF.
- We developed a method with the ability to model and predict seed movements with changing orangutan populations by modelling the ecological cascade of endozoochory mechanistically. This is applicable to the continued study of orangutans at this study site.
- Changes to orangutan population structure and number, particularly female populations, has a potential flow-on effect to floristic composition heterogeneity in TPSF. Furthermore, changes to vegetation structure and productivity may initiate a feedback loop on seed dispersal, since female movement patterns and seed dispersal shadows appear to be dependent upon foraging patterns.
- This is the first objective tool of its kind in orangutan ecological research in TPSF and the first application of T-LoCoH to ecological service provision anywhere. We believe that this process is useful for establishing a training region for mechanistic models to make *a priori* projections of seed dispersal dynamics in novel ecosystems.

## Supplementary Material

Supplementary Data
