# Supplementary material for: An ecophysiologically informed model of seed dispersal by orangutans: linking animal movement with gut passage across time and space
Source: Conserv Physiol. 2018 Mar 28;6(1):coy013. doi: 10.1093/conphys/coy013 (PMC6007347; doi:10.1093/conphys/coy013)
Supplement: Supplementary Data [file coy013tarsziszetal.suppinfo_consphysresubmission.docx]

**Seed mimic passage trials**

Orangutan diet at Taronga Zoo (TZ) was made up to a pre-approved formula which changed for each day of the week but always largely consisted of plant-based material. Food intake could not be quantitatively assessed in this study, however, because all foodstuffs were shared between the two orangutans. The leaf component of the diet offered consisted of whole leaves from the following plants: Celtis (*Celtis australis*); The Weeping Fig (*Ficus benjamina*); Black Mulberry (*Morus nigra*) African Olive (*Olea europaea*) and Banana (*Musa spp*). The vegetables included daily staples of: Sweet potato; spinach; celery; carrot; turnip, lettuce, capsicum and cucumber and a changing roster of fruits that included tomato, kiwi, pear, and apple. Only very small amounts of processed foods were fed, mainly to assist in training and enrichment through a daily ‘activity feed’ of 3 x unshelled peanuts, sultana (10g) and 150g of primate cubes. Water was always freely available at all times. Diets were made up and weighed by dedicated zoo staff, although it could not be distinguished the amounts each orangutan ate of each foodstuff as they shared an exhibit.

**Table S1:** Number of seed mimics of 2, 4 and 6mm ingested by captive orangutans. TZ = Taronga Zoo, PZ = Perth Zoo.

| **Animal** | **2mm** | **4mm** | **6mm** |
| --- | --- | --- | --- |
| TZ Male | 29 | 9 | 6 |
| PZ Male | 45 | 14 | 4 |
| TZ Female | 135 | 16 | 6 |
| PZ Female 1 | 15 | 4 | 3 |
| PZ Female 2 | 19 | 2 | 2 |
| PZ Female 3 | 21 | 12 | 5 |

**Table S2**: Absolute and mean (± SEM) transit times (TT) and maximum transit time (TT_MAX_) for 2, 4, and 6 mm seed mimics in five captive orangutans.

|  | TT (h) | | | TT_Max_ (h) | | |
| --- | --- | --- | --- | --- | --- | --- |
| Animal | **2 mm** | **4 mm** | **6 mm** | **2 mm** | **4 mm** | **6 mm** |
| TZ male | 79.0 | 79.0 | 91.0 | 150.0 | 114.0 | 186.0 |
| TZ female | 58.5 | 41.0 | 19.0 | 119.5 | 67.0 | 43.0 |
| PZ male | 42.0 | 71.0 | 71.0 | 173.0 | 138.0 | 125.0 |
| PZ female1 | 77.0 | 77.0 | 96.0 | 168.0 | 120.0 | 96.0 |
| PZ female 2 | 77.0 | 77.0 | 96.0 | 129.0 | 77.0 | 114.0 |
| PZ female 3 | 90.0 | 90.0 | 144.0 | 216.0 | 192.0 | 144.0 |
| Mean  SEM | **70.6^A^**  **7.1** | **72.5^A^**  **6.8** | **86.2^A^**  **16.6** | **159.3^C^**  **14.2** | **118.0^B^**  **18.4** | **118.0^B^**  **19.6** |

Note: Means with the same superscripts letters are significantly different (*p* < 0.05).
